# Difficult-to-control hypertension: identification of clinical predictors and use of ICT-based integrated care to facilitate blood pressure control

**DOI:** 10.1038/s41371-018-0063-0

**Published:** 2018-05-01

**Authors:** Valeria Visco, Rosa Finelli, Antonietta Valeria Pascale, Pietro Mazzeo, Nicola Ragosa, Valentina Trimarco, Maddalena Illario, Michele Ciccarelli, Guido Iaccarino

**Affiliations:** 1 Department of Medicine, Surgery and Dentistry, University of Salerno, Salerno, Italy; 2 Cardiologia/Utic “PO San Luca”, Vallo della Lucania (SA), Salerno, Italy; 3 Department of Neuroscience, Reproductive and Odontostomatologic Sciences, “Federico II” University of Napoli, Napoli, Italy; 4 Division of Health Innovation, Campania Region Health Directorate, Napoli, Italy

## Abstract

Difficult-to-control (DTC) hypertension represents a burden in real life that can be partially solved through identification of the characteristics of clinical patterns and tailoring antihypertensive strategies, including ICT-enabled integrated care (ICT-IC). In the quest for clinical predictors of DTC hypertension, we screened 482 hypertensive patients who were consecutively referred to the departmental hypertension clinic. Following a data quality check, patients were divided into controlled (C, 49.37%) and uncontrolled (UC, 50.63%) groups based on their systolic blood pressure (BP) at follow-up. We then performed statistical analysis on the demographic, clinical, laboratory, and ultrasound data and observed that older age, female sex, higher BP levels, and a family history of hypertension were predictors of DTC hypertension. We then developed a pilot service of ICT-IC, including weekly home visits by nurses and patient education on self-monitoring of BP, heart rate, body weight, and oxygen saturation using 3G-connected devices. Self-monitored data were transmitted to the hospital servers on the electronic chart of the patient for remote assessment by the hospital hypertension specialists. A total of 20 UC patients (M/F = 10/10; age: 72.04 ± 2.17 years) were enrolled to verify the efficacy of BP control without changes in medical treatment. After 1 month of the ICT-IC program, BP was reduced both at the office assessment (systolic BP (SBP): 162.40 ± 2.23 mm Hg, beginning of the program vs. 138.20 ± 4.26 mm Hg at 1 month, *p* < 0.01) and at home (SBP: 149.83 ± 3.44, beginning of the program vs. 134.16 ± 1.67 mm Hg at 1 month, *p* < 0.01). We concluded that DTC hypertension can be predicted based on the clinical characteristics at the first visit. For these patients, ICT-IC is a feasible therapeutic strategy to achieve BP control.

## Introduction

Despite guidelines, blood pressure (BP) control (<140/90 mm Hg) in hypertensive-treated patients is <40% [1]. The lack of control remains an important issue because a reduction in cardiovascular events cannot be achieved without a reduction in BP [2].

BP control can be considered the collaborative success of at least two partners, namely, the attentive prescribing doctor and constant compliant patient. Reciprocally, lack of control can be considered to be a fault of either one or both partners. Doctors tend to not reinforce the importance of treatment to patients. Additionally, doctors exhibit a resistance to changing the treatment options to a more aggressive approach and opt for an inadequate use of combination therapy [3]. From the patient perspective, the lack of control can be due to poor adherence or reduced compliance as well as to cases of white coat hypertension (approximately 40% of patients with apparent resistant hypertension) [4, 5]. Difficult-to-control (DTC) hypertension includes true resistant hypertension, which is a condition in which BP control is not obtained despite optimal therapy with at least three drugs at the highest tolerated dose [6, 7]. Numerous clinical studies show that a careful evaluation of patient compliance, adequacy of therapy, and lifestyle often reveal evitable contributions to DTC hypertension [6]; however, adherence to therapy remains the main problem, with 50 to 60% of hypertensive patients adhering to therapy [8]. Moreover, on a daily basis, 10% of patients miss a dose of treatment [9, 10], and the number of missing doses increases with the number of prescribed pills [11]. Therefore, when prescribing therapy to hypertensive patients, being aware of the features that identify patients who are at risk for poor control would help the hypertensive specialist tailor strategies of follow-up to avoid failure of therapy.

Intensive follow-up strategies are effective in improving BP control, including the use of electronic charts and networking between general practitioners and hospital specialist centers [12]. Integrated home care represents a further strategy for patients who are at major risk of cardiovascular events. Integrated home care is a health and social integrated care model that is employed to facilitate service users to perform self-care and achieve an independent lifestyle [13]. In integrated care, health and social providers as well as informal caregivers are all connected and integrated around patient needs (food and drug supply, bureaucratic matters as well as the control of dietary and exercise programs, monitoring biometric parameters, drug use, and physical therapy). Information and communication technology (ICT) is proposed to facilitate the exchange of information among the different partners of integrated care and to facilitate patient empowerment by providing patients with the devices that are used for the self-assessment of biomedical parameters [14]. These parameters can, in turn, be remotely monitored by hospital nurses or medical doctors for the care path and/or therapy adjustments.

The present research was therefore undertaken to fulfill two main objectives: first, to identify the predictive features of DTC hypertension at the first patient follow-up; second, to pilot an ICT-enabled integrated home care (ICT-IC) strategy to evaluate its potential in achieving BP control in DTC hypertension patients. To explore the first issue, we performed an observational retrospective analysis of clinical, laboratory, and instrumental data of outpatients at the hypertension clinic at the University Hospital of Salerno Medical School. To test the hypothesis that the use of integrated care would achieve SBP control in these patients without changes in hypertensive medical treatment, we piloted a small study to implement ICT-IC for DTC hypertensive patients.

## Materials and methods

### Recording and organizing the data

The population enrolled in the departmental hypertension clinic was included in a central database (Wincare, TSD-Projects, Milan, Italy), which contained separate electronic sheets for medical history, physical examination, laboratory tests, electrocardiogram, and cardiac and carotid artery ultrasound data. All data were updated at each follow-up visit and stored on the hospital server, protected by a firewall system with user-specific password access.

### Study population

The database of the departmental hypertension clinic at the University Hospital of Salerno Medical School was programmed to identify patients with the following characteristics: essential hypertension; BP sitting >140/90 mm Hg at enrollment; the completion of at least one follow-up visit; completeness of the database; no previous cardiovascular events; no life-threatening comorbidities. The study protocol was approved by the University Hospital Ethical Committee, and informed consent was obtained from all subjects in compliance with the regulations of good clinical practice and privacy. The study population was registered at clinicaltrial.gov (NCT02995954)

### Patient assessment and considered parameters

At enrollment, we collected anamnestic data including the following: family history of hypertension, cardiovascular disease, dyslipidemia, diabetes mellitus (DM) and renal failure; the age at first diagnosis of hypertension and the previous and current pressure values; the previous and current antihypertensive drugs used; any secondary hypertension indicator; the presence of renal disease and/or recurrent urinary tract infections; the existence of thyroid disease; any neurological diseases with particular emphasis to stroke and/or transient ischemic attack; any history of hypertension in pregnancy; taking drugs/substances such as oral contraceptives, liquorice, alcohol, nasal sprays, steroids, and non-steroidal anti-inflammatory drugs; physical activity habits; and a thorough collection of clinical and laboratory parameters and diagnostic tests. At the end of the work-up, optimal medical therapy was prescribed, and a follow-up visit scheduled. At the next follow-up visit, patients received cardiac and carotid artery ultrasounds (Vivid E80, GE Healthcare). Controlled systolic BP (SBP) was defined as sitting SBP <140 mm Hg. In our medical office, BP measurement was obtained with an automated oscillometric BP device (A150 AFIB screen, Microlife AG, Switzerland) that was properly maintained and regularly verified and validated according to international standardized protocols. Cuff placement was preceded by the selection of the appropriate cuff size for the patient’s arm circumference. The lower end of the cuff was placed 2 to 3 cm above the antecubital fossa. The BP reading using the automatic arm BP unit was obtained in the supine, sitting, and standing positions; the cuff was at the heart level in each position of the patient. At least two BP measurements were recorded in every position (spaced 1–2 min apart), and additional measurements were obtained if the first two assessments were largely different. In this study, we considered the average BP in the sitting position with compliance to the current guidelines [15]. The observers were properly trained in the techniques of BP measurement.

### The “Progetto Cuore” calculation of cardiovascular risk

We assessed cardiovascular (CV) risk according to demographic and clinical data based on the charts of the Progetto Cuore of the Italian Institute of Health [16].

### The ICT-IC program

From February 1, 2016 to June 30, 2016, we selected 20 uncontrolled patients, who met the following criteria: (1) poor SBP control (>140 mm Hg after at least three follow-up visits in the last year), (2) optimal drug therapy, including at least three drugs at the maximum tolerated dose, (3) suspicion of poor adherence to therapy, and (4) at least one concurrently treated chronic condition.

Patients who gave informed consent received an ICT-IC program, which included a home care pathway for 4 weeks, with weekly access to a nurse and social caregiver, and telemonitoring of SBP and diastolic BP, heart rate (HR), body weight, body composition % (water and fat), and oxygen saturation using 3G-connected devices. Depending on the level of patient independence, social caregivers provided assistance for the acquisition of food and drug supplies, bureaucratic matters as well as the control of dietary and exercise programs. Nurses provided personalized assistance to the patients in terms of monitoring biometric parameters, drug use, and physical therapy.

Patients who received the ICT-IC home care program were instructed to use of 3G-connected devices. They measured their BP twice in the morning and twice in the evening with an automated oscillometric BP device (Seniorlab, Cygnus, Germany) connected via Bluetooth with a smartphone for data transmission over the 3G/4G data transmission network. The lower end of the appropriate cuff was placed 2 to 3 cm above the antecubital fossa, and the BP reading was obtained in a sitting position (after a resting period of 5 min) with the cuff at the heart level. If a patient was unable to attend to these tasks, their caregiver (relative, volunteer, informal caregiver) was trained to use the devices. Each patient performed at least a scheduled (once a week) self-assessment of the above parameters. On the day of their access to the home of the patient, nurses assessed patient hemodynamics, clinical parameters, and completed quality-of-life questionnaires. Treatment compliance was verified weekly by the nurse through pill count. Data were securely transmitted through the 3G/4G with an encryption data transmission protocol to the hospital servers in Salerno and were accessible through the means of a web-based client. After 4 weeks, patients were evaluated on the hospital premises.

### Statistical analysis

Data were checked for quality to exclude patients whose data needed to implement the logistic regression were missing. The independent variables considered in this study included the following: sex; age; body mass index (BMI); smoking status; physical activity habits; diagnosis of DM; family history of high BP; number of years since diagnosis of hypertension; previous SBP referral in the absence of any therapy; SBP measured in the sitting position, pulse pressure (PP), HR, ankle-brachial pressure index (ABI), and New York Heart Association class at the first visit; CV risk; class of drug taken (diuretics, β-blockers, α-blockers, renin–angiotensin system (RAS) inhibitors, and dihydropyridine calcium channel blockers); blood glucose, triglycerides, total cholesterol, high-density lipoprotein (HDL), and low-density lipoprotein (LDL) calculated using the appropriate formula; left ventricular mass (LVM) index and E/A ratio; presence of plaques in the carotid arteries; and rapidity of the first follow-up visit (the time interval of the first follow-up is defined retrospectively as the interval, in days, between the first and the second visit).

The data are expressed as frequencies and percentages for qualitative variables and as the means ± SEM for quantitative variables.

For the ICT-IC study, we identified the sample size needed to observe a reduction in SBP by 15 mm Hg with an *α*-value of 0.05 and a power of 90%. The result of the analysis showed that a sample size of 17 patients was needed.

Each patient represented his/her own control using a paired statistical design. Additionally, data from the two visits prior to the last visit were used as a standard care control.

The association between categorical variables was assessed using the *χ*^2^test. The statistical significance of the means was calculated using the *t* test or analysis of variance as appropriate. *P* < 0.05 was considered statistically significant.

Significant parameters were further analyzed using a logistic regression to determine the clinical variables associated with the non-achievement of the target BP; for the selection of variables, the stepwise backward method was used. The statistical analysis was performed using SPSS software for Windows, version 22.0 (SPSS, Inc., Chicago, IL, USA).

## Results

Among the 482 hypertensive patients referred to the outpatient clinic, we identified 156 patients with uncontrolled BP at the first visit. After data quality control and assessment, 79 patients were included in the analysis. Based on the achievement of the target systolic pressure by the first available follow-up visit, this population was divided into two subgroups, namely, uncontrolled patients (UC, *n* = 40, 50.63%) and controlled patients (C, *n* = 39, 49.37%). The clinical features at first visit for both groups are reported in Tables 1 and 2. To predict SBP control at the first follow-up visit, we compared clinical, laboratory, and instrumental parameters and therapy between the two groups.

**Table 1:** Clinical characteristics of selected population

Variables	Total population (*n* = 79)		Controlled (C) (*n* = 39)		Uncontrolled (UC) (*n* = 40)		*p* value
		%		%		%	
Sex							<0.001
M	36	45.6	23	58.9	13	32.5	
F	43	54.4	16	41.1	27	67.5	
Age (years)	62.6 ± 1.3		59.6 ± 1.8		65.6 ± 1.9		0.023
BMI (kg/m^2^)	29.5 ± 0.6		29.3 ± 0.9		29.7 ± 0.9		NS
Hypertensive father	28	35.4	10	25.64	18	45	0.016
Hypertensive mother	35	44.3	19	48.7	16	40.0	NS
Diabetes	10	12.7	3	7.7	7	17.5	<0.01
Smoking status	24	30.4	13	33.3	11	27.5	NS
Physical activity	12	15.2	10	25.6	2	5.0	<0.001
Years of HBP	9.0 ± 1.0		7.3 ± 1.2		11.0 ± 1.6		NS
SBP before any therapy (mm Hg)	160.7 ± 2.5		154.9 ± 3.2		166.4 ± 3.6		0.020
SBP (mm Hg)	157.4 ± 1.5		154.31 ± 2.06		160.5 ± 2.2		0.045
Pulse pressure (mm Hg)	64.9 ± 1.6		61.4 ± 2.2		68.4 ± 2.1		0.023
HR (bpm)	72.5 ± 1.8		74.9 ± 2.9		70.2 ± 2.1		NS
ABI	1.29 ± 0.02		1.27 ± 0.02		1.31 ± 0.00		NS
Follow-up timing ≤90 days	52	65.8	21	53.9	31	77.5	<0.001

Data are presented as means ± SEM, unless otherwise indicated

*BMI* body mass index, *HBP* high blood pressure, *SBP* systolic blood pressure, *HR* heart rate, *ABI* ankle-brachial index, *NS* not significant

**Table 2:** Laboratory, US parameters, and therapy of selected population

Variables	Controlled (C)		Uncontrolled (UC)		*p* value
		%		%	
T-CHOL (mg/dl)	195.05 ± 6.03		195.30 ± 7.63		NS
HDL low	3	7.69	13	32.50	0.001
LDL (mg/dl)	111.50 ± 5.95		115.59 ± 6.96		NS
Triglycerides (mg/dl)	138.28 ± 9.51		146.60 ± 11.46		NS
Glycemia (mg/dl)	105.54 ± 5.42		105.80 ± 4.02		NS
LVH	18	46.15	16	40	NS
LVMI (g/m^2^)	118.93 ± 7.28		120.51 ± 11.42		NS
Carotid plaques	16	41.03	16	40.00	NS
Carotid IMT max (mm)	1.78 ± 0.16		1.87 ± 0.19		NS
E/A	0.93 ± 0.06		0.84 ± 0.04		NS
Combination therapy	15	38.46	25	62.50	0.002
Diuretics	16	41.03	26	65.00	0.002
β-blockers	23	58.97	22	55.00	NS
RAS inhibitors	32	82.05	40	100.00	NS
Dihydropyridine calcio-antagonists	13	33.33	21	52.50	0.018

Data are presented as means ± SEM, unless otherwise indicated

*T-CHOL* total cholesterol, *HDL* high-density lipoprotein, *LDL* low-density lipoprotein, *LVH* left ventricular hypertrophy, *LVMI* left ventricular mass index, *IMT* intima–media thickness, *combination therapy* use of at least two antihypertensive drugs, *RAS* renin–angiotensin system; *NS* not significant.

### Clinical parameters

The two groups differed in sex, age, SBP before any therapy, SBP at the first visit, PP, family history of paternal hypertension, physical activity, diabetes, and time to the first follow-up visit (≤90 days). No difference was observed in BMI, family history of maternal hypertension, smoking status, years of high BP, HR, and ABI (Table 1).

### Laboratory parameters

The results showed that the patients in the UC group had significantly lower HDL levels (Table 2), considering a cutoff value of 46 mg/dl for women and 40 mg/dl for men as suggested by the ESH guidelines/ESC 2013. Total cholesterol, LDL, triglyceride, and glucose were similar between the two groups.

### Cardiac and vascular ultrasounds

The two groups did not differ in ultrasound-assessed cardiac and vascular target organ damage (Table 2); in particular, they were similar in the LVM index, E/A ratio, and maximum carotid IMT.

### Pharmacological therapy

The two groups were comparable according to pharmacological therapy. As reported in Table 2, there was no difference in drug numbers. Differences emerged in the use of some pharmacological classes; for example, the UC patients more often received diuretics and dihydropyridine calcium channel blockers. No difference was observed between the two groups for the prescription of β-blockers and RAS inhibitors.

### Predictors of SBP control at follow-up

According to the logistic regression analysis using a stepwise backward method of the significant parameters, the major predictors of therapeutic success were age, sex, SBP at the first visit, family history of arterial hypertension on the paternal side, and HDL (Table 3).

**Table 3:** Logistic regression

Step 4	Wald	Sig.	Exp(*B*)
SBP	6.687	0.010	0.943
Hypertensive father	5.630	0.018	5.220
Age	9.127	0.003	0.913
HDL	9.764	0.002	24.377
Sex	3.874	0.049	3.424

*SBP* systolic blood pressure, *HDL* high-density lipoprotein

### Achievement of target BP through the Beyond Silos home care program

**Fig. 1:**
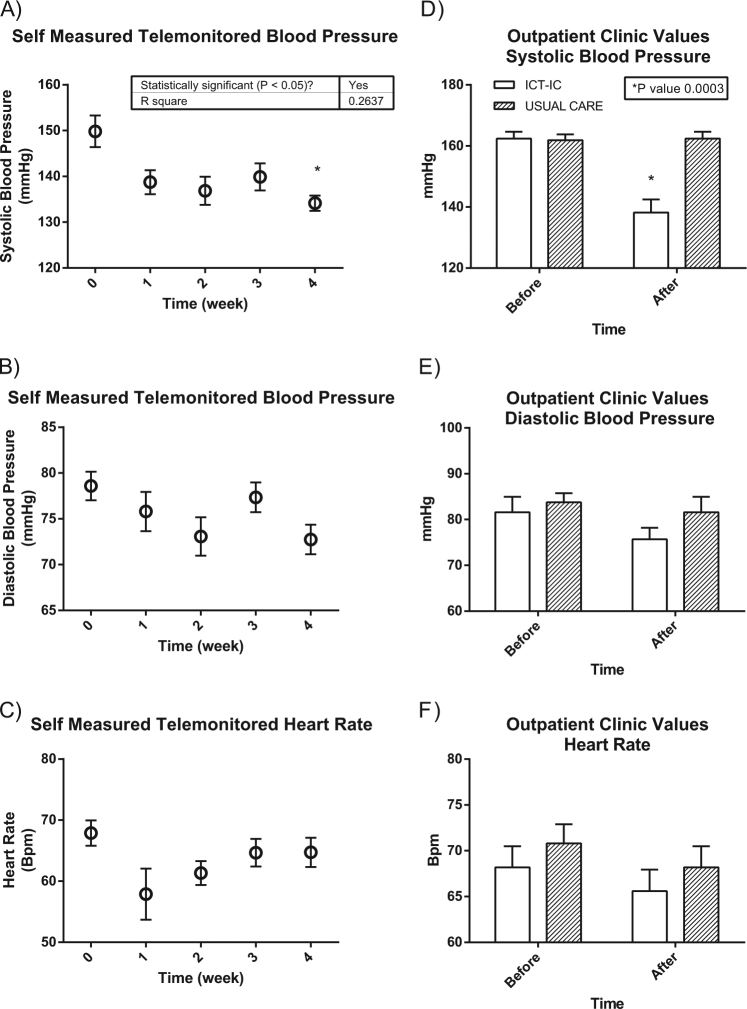
Blood pressure and heart rate values telemonitored weekly (**a**–**c**) or measured before and after the intervention in the office (**d**–**f**). The self-measured telemonitored SBP was significantly different before and after the program, and this was true also for SBP in the office (**a**, **d**) (*p < 0.05). No difference was observed for the same patients who maintained follow-up for usual care

A total of 20 patients from the UC group were enrolled in the ICT-IC program “Beyond Silos.” The clinical features of these patients are reported in Table 4. In this population, BP decreased both at home and in the office, while no changes were observed in HR. In particular, SBP in the office was significantly different before and after the program (before vs. after; SBP: 162.40 ± 2.23 vs. 138.20 ± 4.26 mm Hg, respectively, *p* < 0.01) and this was true also for self-measured telemonitored SBP (before vs. after; SBP: 149.83 ± 3.44 vs. 134.16 ± 1.67 mm Hg, respectively, *p* < 0.01) (Fig. 1).

**Table 4:** Clinical characteristics of the 20 subjects that received ICT-IC home care program

Variables	*n*	%
Sex (M/F)	10/10	50.00/50.00
Age (years)	72.04 ± 2.17	
Weight (kg)	81,80 ± 3.75	
Height (cm)	164.10 ± 2.15	
BMI (kg/m^2^)	30.17 ± 0.95	
HR before the program (bpm)	68.20 ± 2.29	
SBP before the program (mm Hg)	162.40 ± 2.23	
DBP before the program (mm Hg)	81.60 ± 3.35	
Glycemia (mg/dl)	112.5 ± 4.66	
Hypercholesterolemia	10	50.00
HDL (mg/dl)	54.00 ± 3.03	
Physical activity	0	0.00
Smoking status	4	20.00
Diabetes	6	30.00
Years of hypertension	15.43 ± 2.78	
Combination therapy	20	100.00
History of CVE	8	40.00

*ICT-CT* ICT-enabled integrated care, *BMI* body mass index, *HR* heart rate, *SBP* systolic blood pressure, *DBP* diastolic blood pressure, *HDL* high-density lipoprotein, *CVE* cardiovascular events, *combination therapy* use of at least two antihypertensive drugs

**Fig. 2:**
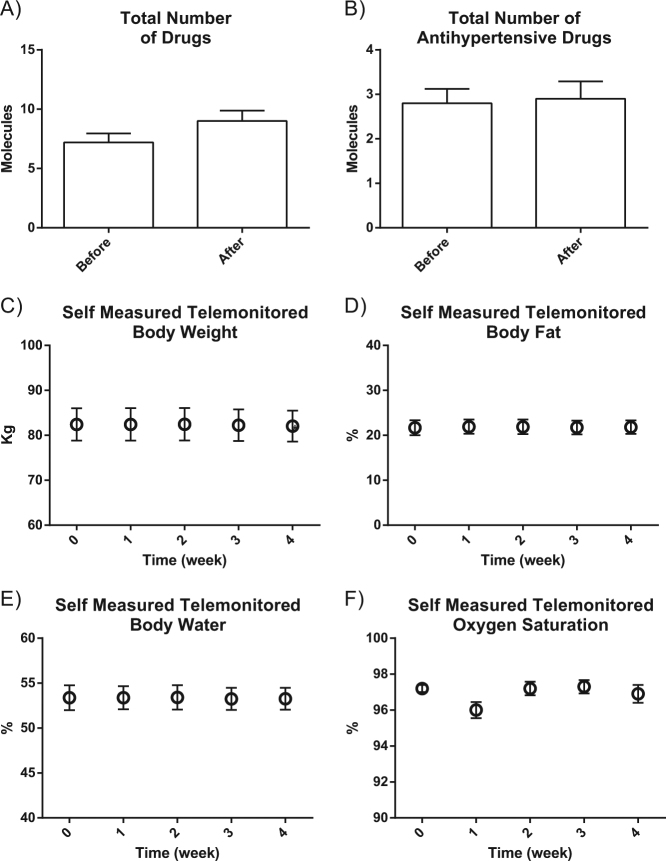
Number of total compounds and types of antihypertensive drugs and other weekly telemonitored parameters in hypertensive patients before and after the ICT-IC home care program is shown. The graphs show that no changes were observed in the number of total drugs (A), antihypertensive drugs (B), Body weight (C), Body composition (D and E) and oxygen saturation (F).

**Fig. 3:**
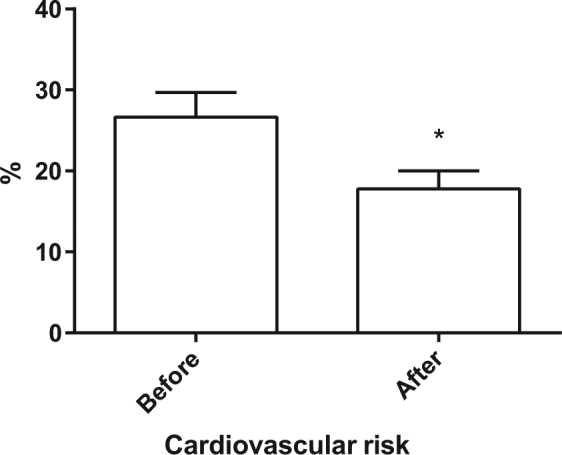
CVR before and after 1 month of intervention. Before the ICT-IC home care program (left graph), cardiovascular risk was statistically significantly higher than after the program (right graph) (*p < 0.05)

This effect was specific to the ICT-IC intervention because no reduction in BP had been observed in the same patients when receiving the usual care (Fig. 1). Other monitored parameters did not change during the period of observation, including body weight and oxygen saturation (Fig. 2). Interestingly, there was a statistically significant reduction in CV risk measured according to the Progetto Cuore [16] after 4 weeks of the ICT-enabled program (Fig. 3). This result was achieved without changes in prescription patterns (Fig. 2) and drug dosages (data not shown).

## Discussion

The new paradigms for medical care strive for precision and are all based on patient stratification and classification. Therefore, it becomes important to study available databases that include clinical data of patients and to profile patients who are in need of personalized therapeutics. This is particularly true for chronic conditions, such as hypertension, that affect large segments of the general population. For this condition, in particular, the failure rate of optimal BP control in treated patients is still too high, contributing to unbearable morbidity and mortality rates [1, 2]. Therefore, the early identification of patients who are not going to have successful outcomes from therapy is important for the management of hypertension from a public health perspective. Through a retrospective analysis of a large database, the purpose of this study was to identify the factors that contribute to a lack of control of SBP. Several studies have shown that the achievement of SBP control is less common than the achievement of the diastolic target [17–19]. In this paper, we demonstrated that it is possible at the first visit to profile those hypertensive patients who will develop DTC hypertension.

The results of this research offer the opportunity to think about the determinants of failed SBP control. First, our analysis showed that the control of SBP was the most difficult to treat in older patients. This observation is in agreement with the literature, according to which subjects aged 40 to 60 years attained a better BP control than older patients (≥60 years) [20]. In our population, the control of SBP was more difficult in women. Regarding this issue, Gu et al. [21] reported that among hypertensive patients, despite a larger use of antihypertensive drugs (in particular, diuretics and angiotensin receptor blockers) in women (61.4% vs. 56.8% in men), only 44.8% of women attained the target pressure vs. 51.1% of treated men. This suggests that hypertensive women are less likely to attain BP control than men.

Among the mechanisms underlying this observation, different genetic backgrounds should be considered. It is known that X-linked variability participates in the development of the hypertensive state [22]. Indeed, included among the major predictors of a lack of BP control at follow-up is the maternal transmission of hypertension [22].

Physical inactivity is one of the predictors of poor control of SBP. In our database, this demographic report is based on self-declared activity; nevertheless, physical inactivity is also confirmed by lower HDL levels observed among the uncontrolled hypertensives in our database. Indeed, previous reports showed that the beneficial effects on cardiovascular risk induced by physical activity are at least in part explained by the increase in HDL levels [23]. Additionally, the changes in HDL induced by exercise may be sex dependent; the volume of exercise needed to raise HDL levels is significantly higher for women than men [23].

SBP levels at the first visit is another important predictor of poor SBP control. Patients with a higher degree of hypertension are less controlled at the next follow-up visit. Guidelines and clinical experience suggest a combined therapy approach at the first visit for patients with moderate and severe hypertension [15, 24]. Our analysis showed that closer follow-up visits, especially for patients most at risk for DTC hypertension, will lead to better management of therapy and comorbidities. Nevertheless, it is not always possible to increase the frequency of hospital or office access. Therefore, a home care service, supported by telemonitoring and ICT-based transmission of remotely measured physiological parameters, can be considered to improve BP control. Our data showed that patients with a history of poor BP control despite optimal therapy can achieve control through a digitally supported home care program within a month. This suggests that strategies of ICT-based home care might improve the effectiveness of self-monitoring and encourage adherence to the prescriptions with the aim of increasing the rate of hypertension control, in particular for patients with multiple morbidities and poor compliance. These systems provide complete information with high reliability, ease of use and speed. In agreement with these observations, the e-Health for Safety document published by the European Commission in 2007 (review.epractice-en/en/library/302671) highlighted the impact of ICTs on risk management and patient safety. Evidence is mounting that ICT in medicine can help to prevent medical errors and adverse events, initiate rapid response to an event (allowing its monitoring), provide information that can facilitate diagnostic and therapeutic decisions, encourage patient involvement in decision-making with a lead in terms of compliance, and decrease patient management costs [15, 25]. Furthermore, many clinical studies show that transmission by telemetry of the home-BP measurements offers good support for BP control [26, 27]. In our study, we achieved BP control within 1 month of ICT-IC. This evidence is contrary to the ineffective outcome of the very same therapy in patients over an extended period of time (at least 12 months, by inclusion criteria). The reason for this quick attainment of BP control is the better adherence of patients to treatment as a consequence of digital assistance during their daily life, leading to a better perception of their condition and subsequent empowerment. Our study lacks data regarding the long-term effects of this intervention; therefore, we cannot exclude a Pokemon-Go effect, that is, a loss of interest in the technology by a user that returns to their usual behavior [28]. Nevertheless, the intervention is feasible and repeatable. Furthermore, our study was performed year round, and different patients received ICT-IC at different times of the year; thus, we can exclude any effect of time, weather, season, or vacation as being responsible for the beneficial effects observed after ICT-IC [28]. Studies that have employed communication technologies showed that there are many new ways to communicate with patients, with theoretical advantages in terms of time and improvement in therapeutic plans. As some studies have demonstrated, telemonitoring home-BP is an example of how electronic transmission of the self-measurement of BP can lead to better adherence to treatment and more effective BP control [29, 30]. However, ICT per se is not enough to empower the patient. In our design, a critical role was played by the nurses and social workers who provided patients with personalized care, explained the importance of therapy and lifestyles, and taught the patients to take good care of themselves. We believe that such activities had a relevant impact on our results.

BP control is excessively low in the hypertensive population, and in our country, it does not exceed 40%, resulting in only partial effectiveness of prevention strategies. The improvement in BP control is a very important element in reducing the burden of disease related to uncontrolled HBP and its health-related costs. The hypertension management cost in the period preceding the development of organ damage and major CV events was significantly lower compared to the social and health costs linked to the consequences of poorly controlled hypertension.

The knowledge of the determinants of SBP control can undoubtedly increase the chance and percentage of achieving predetermined targets. In particular, our data indicated that therapeutic success in the first follow-up was largely dependent on anthropometric factors related to the characteristics of the patient, such as age, gender, family history, and degree of hypertension, which are risk factors that cannot be modified and that can allow us to predict therapeutic success at the first visit.

Exercise and control of other risk factors, including dyslipidemia, are associated mechanisms that can be corrected using appropriate and effective measures.

The strategies and interventions that can synergistically act on the multiple determinants of poor and inadequate BP control include the organization of the follow-up network in clinical and non-clinical fields; establishment of communication between specialist centers, general practitioners and patients; and based on patient empowerment, if the patient becomes an active part in managing the disease.

Strategies of ICT-based home care might represent a real breakthrough in the management of chronic conditions, in particular, for patients who have multiple morbidities and poor compliance. Future large-scale studies are needed to assess the long-term effects on cardiovascular outcomes.

### Summary Table

#### What is known about the topic?

- Despite guidelines, actual BP control in treated hypertensive patients is <40%.
- Intensive follow-up strategies are effective in improving BP control, including the use of electronic charts.

#### What this study adds?

- Difficult-to-treat hypertension can be predicted based on clinical features.
- The use of ICT-IC is a personalized strategy of therapy that can preventively be applied in patients who present features of DTT hypertension at the first visit.

## Electronic supplementary material

Supplement
