# Supplementary material for: Difficult-to-control hypertension: identification of clinical predictors and use of ICT-based integrated care to facilitate blood pressure control
Source: J Hum Hypertens. 2018 May 1;32(7):467–76. doi: 10.1038/s41371-018-0063-0 (PMC6057905; doi:10.1038/s41371-018-0063-0)
Supplement: Supplementary file 1 — Supplement [file 41371_2018_63_MOESM1_ESM.doc]

**SPECIFICATIONS OF AUTOMATED OSCILLOMETRIC BP DEVICE (SENIORLAB, CIGNUS, GERMANY)**

**System Performance**

Power Source: Four 1.5V AA alkaline batteries

Size the monitor w/o cuff: 140 (L) x 120 (W) x 70 (H)mm, 350g without batteries.

Cuff Size: M (medium): 24-35 cm (9.4-13.8 inches); XL (large): 24-43 cm (9.4-17 inches)

Memory: Maximum 400 memory records

Power saving: Automatic power off if system idle for 3 minutes.

System operating condition: 10°C - 40°C (50°F-104°F), below 85% RH

Meter storage condition: -20°C – 60°C (-4°F – 140°F), 5-95% RH

Power Supply Input: DC +6V / 1A (max) via Power Plug

**BP measurement performance**

Pressure range: 0-300 mmHg

Heart rate range: 40-199 beat per minute

Systolic Measurement Range: 50-250 mmHg

Diastolic Measurement Range: 30-180 mmHg

Pulse Rate Measurement Range: 40-199 beats/minute

Maximum inflation pressure: 280 mmHg

Accuracy of Pressure: ±3 mmHg or ±2% of reading

Accuracy of Pulse rate: ±4% of reading

Measurement unit: Either mmHg or Kpa

This device has been tested to meet the electrical and safety requirements of: IEC/EN 60601-1, IEC/EN 60601-2.

**Reference to Standards:**

- EN 1060-1 /-3, NIBP-requirements
- IEC60601-1 General requirement for safety
- IEC60601-1-2 Requirements for EMC
- EN1060-4, NIBP clinical investigation
- ANSI/AAMI SP10, NIBP requirements
